# A Pathological Fracture of the Femoral Neck Revealing Villonodular Synovitis

**DOI:** 10.7759/cureus.68278

**Published:** 2024-08-31

**Authors:** Achraf Tebbaa El Hassali, Mohammed Barrached, Adnane Lachkar, Najib Abdeljaouad, Hicham Yacoubi

**Affiliations:** 1 Orthopedics and Traumatology, Faculty of Medicine and Pharmacy of Oujda, Mohammed VI University Hospital, Mohamed I University, Oujda, MAR

**Keywords:** pain, tumor, villonodular, synovitis, pathological, fracture

## Abstract

Villonodular synovitis is a rare disease of the synovial tissue occurring most commonly in synovial joints such as the knee and ankle joints. We report the case of a patient presenting with villonodular synovitis of the hip revealed by a pathological fracture of the femoral neck, discussing our diagnostic and therapeutic approach with recent scientific data.

## Introduction

Villonodular synovitis is a rare disease of synovial tissue occurring most commonly in the synovial joints, such as the knee and ankle joints [1]. Apart from synovial joints, all synovial structures, such as tenosynovial tissue and the bursa, can in principle be affected [2]. The knee is the joint most affected by this disease, which is observed in young adults but also in children [3]. The diagnosis can be suspected on MRI, but only a biopsy can confirm it. The treatment is essentially surgical for the symptomatic and diffuse form.

We report the case of a patient presenting with villonodular synovitis of the femoral neck revealed by a pathological fracture, discussing our diagnostic and therapeutic approach with recent scientific data.

## Case presentation

We report the case of a 63-year-old patient, a housewife with no notable personal or family pathological history. The patient consulted for pain in the left hip that had been present for six months without any notion of trauma. Initially, she consulted her family doctor who put her on paracetamol of 3 g/day and a nonsteroidal anti-inflammatory drug without improvement.

The pain was progressive, of moderate intensity (5/10 visual analog scale), intermittent, and then became permanent, without irradiation and of an inflammatory type. This clinical picture worsened a month before by the increase in the intensity of the pain and the onset of total functional impotence of the left hip.

The clinical examination found a conscious patient, hemodynamically and respiratory stable and in good general condition: heart rate at 86 beats per minute, blood pressure at 120/68 mmHg, respiratory rate at 24 cycles per minute, temperature at 37°C, body mass index at 19.5 kg/m^2^.

During the osteoarticular examination, the patient was lying down, unable to lift the heel of the left foot from the examination table. The antero-external aspect of the left hip is the site of painful swelling, of hard consistency, and adherent to the deep and superficial planes. Active and passive mobilization is impossible because of pain. The vascular and nervous examination was unremarkable, and the ganglionic areas were free. Examination of the rest of the left limb and the contralateral limb was without abnormality.

**Figure 1:**
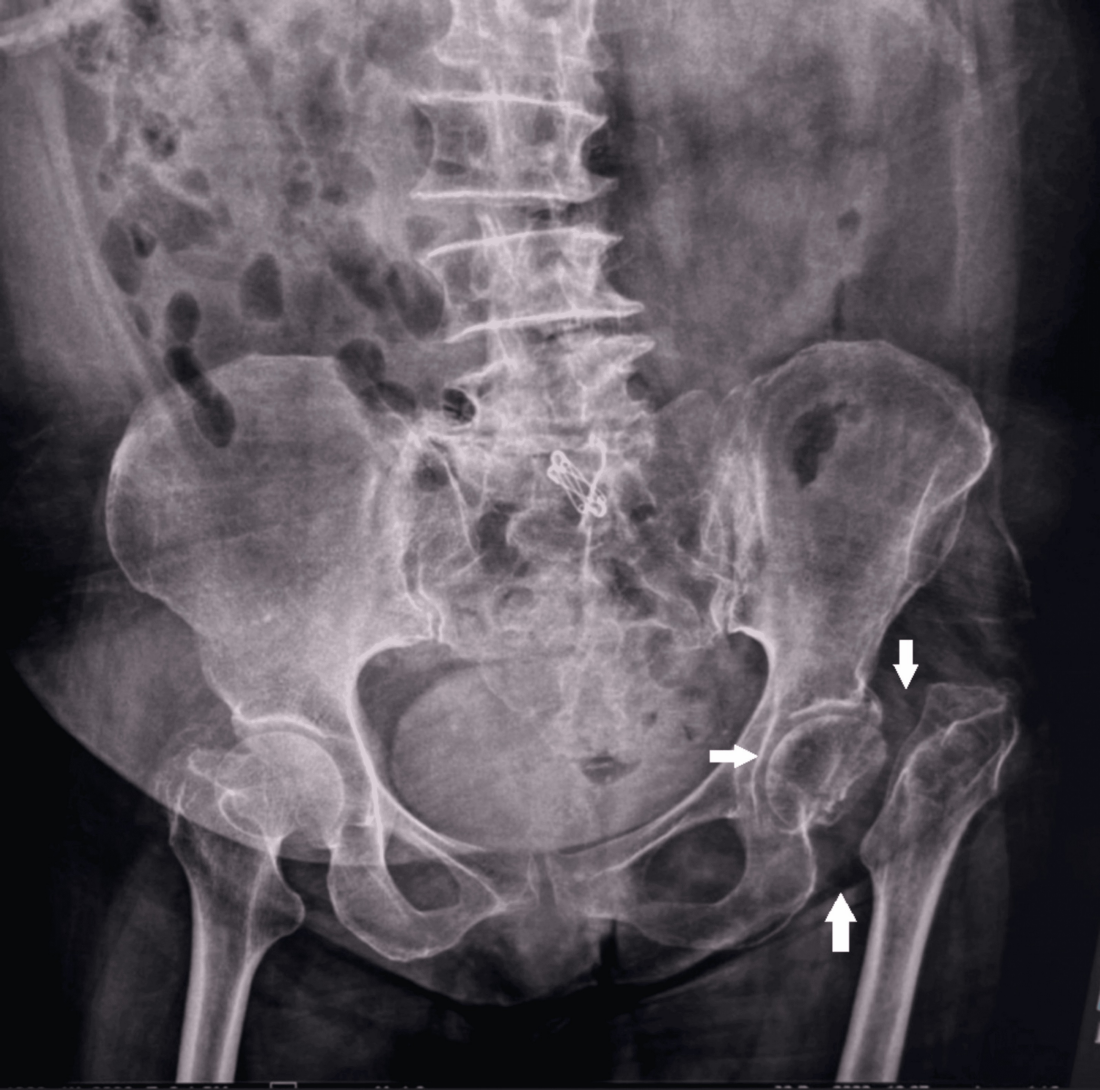
A standard frontal X-ray of the pelvis showing the pathological fracture of the left femoral neck

**Figure 2:**
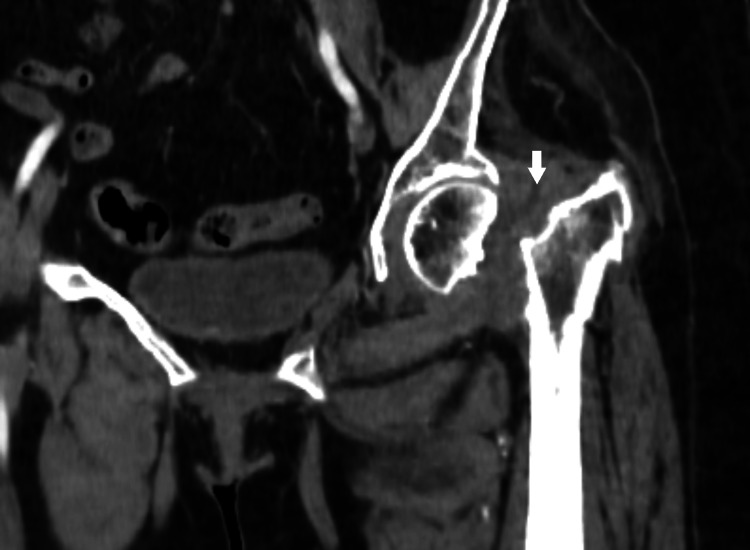
A non-contrast CT scan of the left hip in the front section showing the lesion

**Figure 3:**
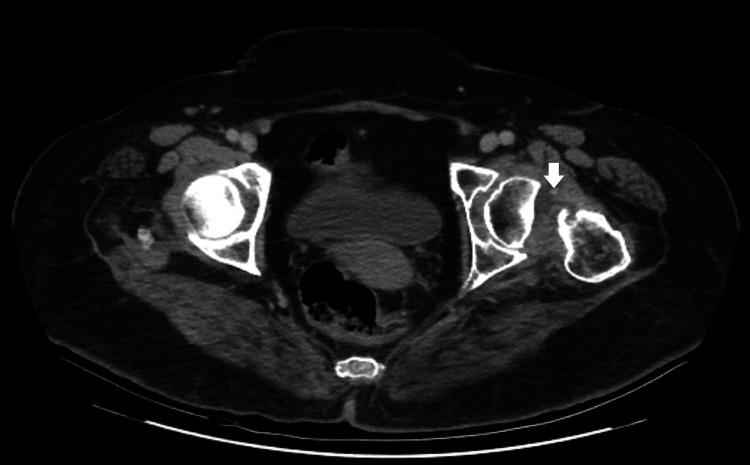
A non-contrast CT scan of the left hip in the axial section showing the lesion

The patient underwent a standard frontal X-ray of the pelvis before and after treatment and a CT of the pelvis revealing a lesion process in the soft parts of the left femoral neck, poorly defined, heterogeneous, and isodense compatible with a tumor process associated with a fracture and lysis of the left femoral neck (Figures 1-3).

**Figure 4:**
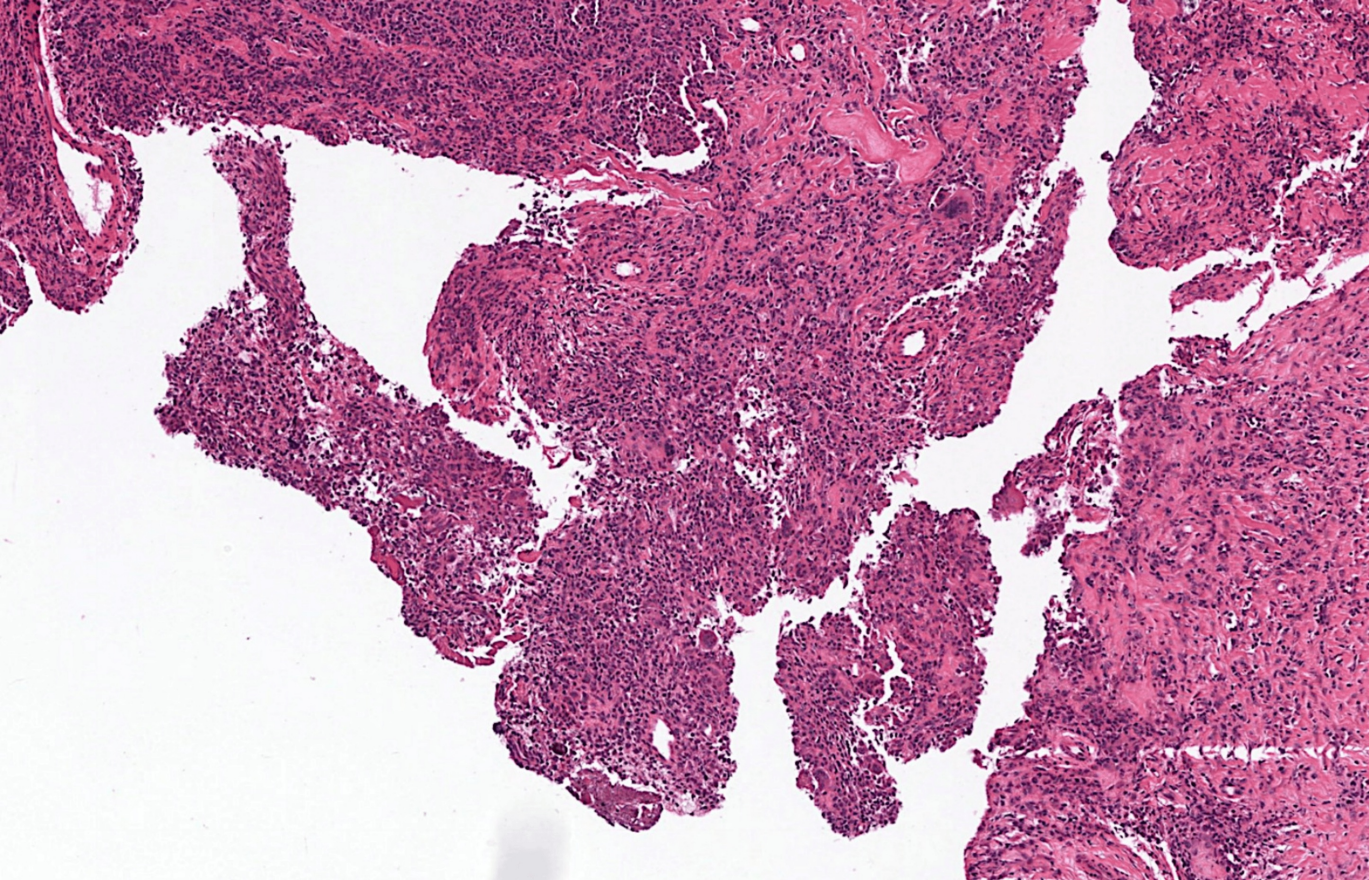
Photomicrograph of the lesion showing a papillary proliferation of synovial tissue (A: H&E, x40)

For diagnostic confirmation, the patient underwent a surgical bone and soft parts biopsy of the left femoral neck with anatomopathological analysis. The latter came back in favor of a remodeled villonodular synovitis with no signs of malignancy (Figure 4).

**Figure 5:**
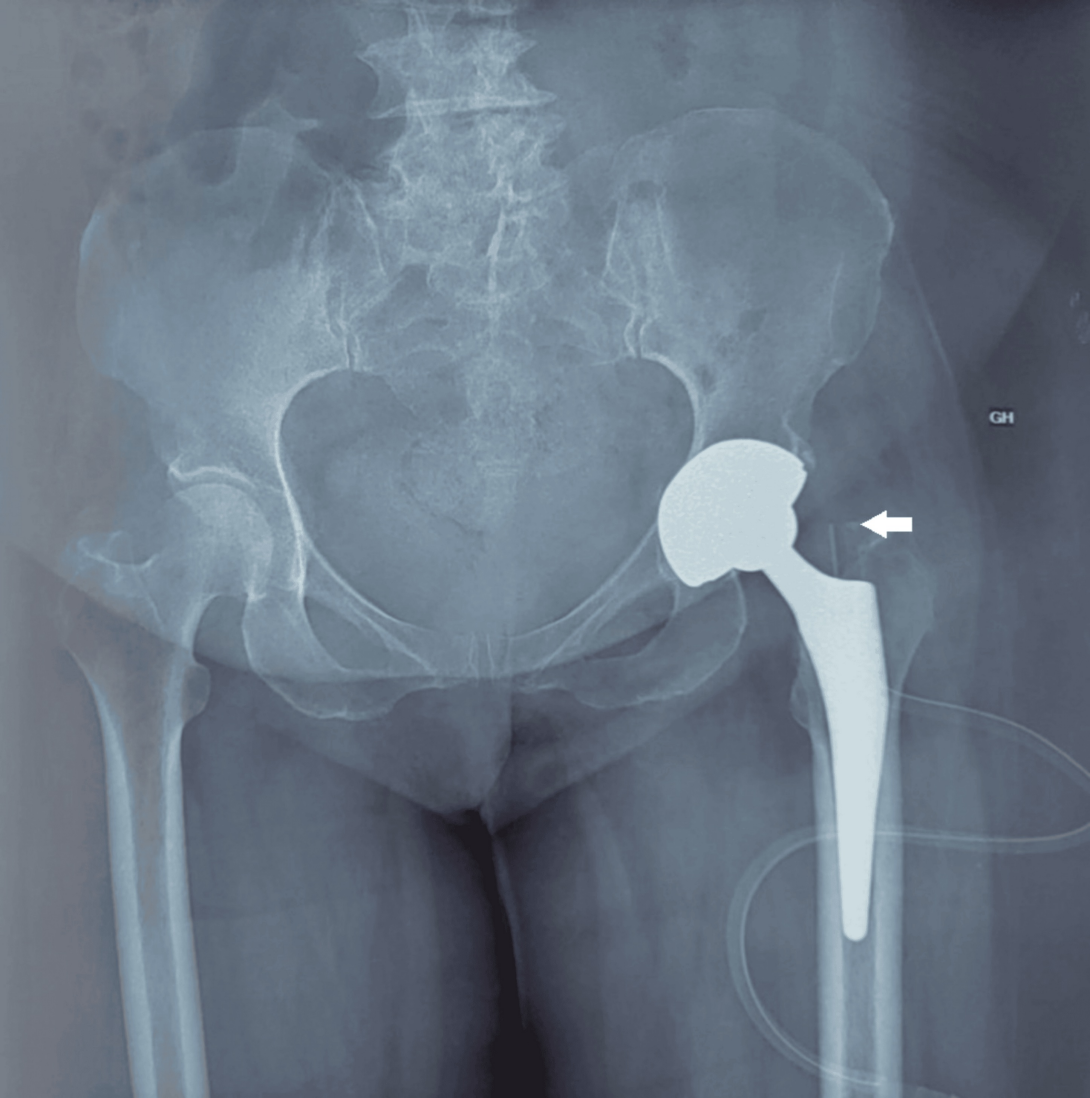
Postoperative standard frontal X-ray of the pelvis

The patient underwent a synovectomy with a total left hip prosthesis replacement. Regular clinical and radiological (X-ray) follow-up of the patient for 12 months has not revealed any recurrence with a clear clinical and functional improvement (Figure 5).

## Discussion

The first description of villonodular synovitis dates back more than a century in 1852. Its incidence is approximately 1.8/million person-years, and it often affects adults aged between 30 and 40 years old with a female predominance [4,5]. Rare cases of villonodular synovitis in subjects aged over 50 such as our patient have been reported [6].

Classically the pathology is single joint limited to the knee, but cases of polyarticular villonodular synovitis or in unusual locations such as the finger or temporomendibular joints have been reported [7,8].

Villonodular synovitis is characterized by a benign proliferation of the synovium. Its clinical presentation is not very specific and variable. Patients often consult for the appearance of arthralgia with functional impotence and joint swelling, but the diagnosis is often made late [9]. Early diagnosis improves prognosis and helps avoid complications.

Histologically, we distinguish two forms with a prognostic difference, and there is also an extra-articular form: (1) tenosynovial giant cell tumor (TGCT), which is a localized form, rare, and not very recurrent after surgical treatment, and (2) diffuse-type giant cell tumor (Dt-GCT) or pigmented villonodular synovitis (PVNS), which is a diffuse and very recurrent form after surgery.

Histologically, villonodular synovitis presents in the form of a collagen stroma associated with a cell mass that can variably include a wide variety of cell types, such as macrophages often loaded with hemosiderin, histiocytes, multinucleated giant cells, and xanthomatous cells. Chronic nonspecific lymphocytic inflammation is also often found [10].

The exact pathophysiology of this disease remains poorly understood; however, two hypotheses have been proposed. The first considers villonodular synovitis as an inflammatory pathology reaction to bleeding or repeated trauma. The second suggests a monoclonal tumor proliferation involving several genes [10].

Standard radiographs can show secondary degenerative disorders depending on the site of the villonodular synovitis and can guide therapeutic management. The presence of pseudocysts at the coxofemoral level outside the regions of pressure (femoral neck, acetabulum) should raise suspicion of villonodular synovitis. MRI is the radiological modality of choice in cases of clinical suspicion of villonodular synovitis, but diagnostic confirmation is histological on synovial biopsy [11].

Therapeutic management is guided by the clinical form. In the localized form, a synovectomy, whether by arthroscopy or arthrotomy, is sufficient while in the diffuse form surgery, it can be associated with brachytherapy, external radiotherapy, or the use of a tyrosine kinase inhibitor such as imatinib (Glivec) [12,13].

The particularity of our case lies in the unusual age of onset of the disease and its location and its consequences. We believe we have not found any similar cases. A prosthetic replacement was the only possible option to replace the weakened bone and allow clinical and functional recovery despite surgical resection of villonodular synovitis.

## Conclusions

Villonodular synovitis is a rare disease of young adults often overlooked. Its recurrent nature can make its management particularly difficult. Early diagnosis improves the prognosis and helps avoid complications.
